# Supplementary material for: Multi-tissue network analysis reveals the effect of JNK inhibition on dietary sucrose-induced metabolic dysfunction in rats
Source: eLife. 2025 Feb 11;13:RP98427. doi: 10.7554/eLife.98427 (PMC11813226; doi:10.7554/eLife.98427)
Supplement: Supplementary file 9. [file elife-98427-supp9.docx]

Table 1. Metabolites associated with sucrose overconsumption in MASLD.

| **Study** | **Intervention(s) and duration** | **Metabolites** | **Metabolism** |
| --- | --- | --- | --- |
| Fujii et al., 2024 [1]. | Male rats fed a 10% sucrose solution for 16 days | Decreased blood acetate and butyrate levels | Gut microbial metabolism, Lipid metabolism |
| Sun et al., 2021 [2]. | Male Wistar rats fed high-sucrose diet for 4 weeks | Decreased butyrate and formate in the cecal content |  |
| Song et al., 2024 [3]. | Male Wistar rats fed a high sucrose diet for 4 weeks | Decreased acetate and butyrate; increased succinate in cecal content |  |
| Ramos-Romero et al., 2019 [4]. | Male Wistar rats were fed a 35% sucrose solution for 24 weeks. | Increased uric acid in urine | Urea cycle |
| Ortiz & Field, 2023 [5]. | Male C57BL/6J mice were fed either low-fat diet or high-fat diet with 30% sucrose water for 8 weeks | Elevated serum and kidney erythritol concentration | Carbohydrate metabolism |
| Beckmann et al. 2015 [6]. | Healthy females (n=90) consumed 0, 50, or 100g sucrose in water, followed by urine and blood sampling at 0, 3, and 24 hours. | Increased erythronic acid in plasma | Carbohydrate metabolism |
| He et al., 2023 [7]. | Male C57BL/6J mice were fed a high sucrose diet (30% sucrose in drinking water) for 24 weeks | Decreased muricholic acid level in the liver, cecal and colon content. increased hyocholic acid levels in the serum. | Bile acid metabolism |
| Stephenson et al. 2022 [8]. | Mice fed 10% sucrose solution in drinking water for 12 weeks | Increased triglyceride-bound oleate, palmitate, and stearate in liver; Mixed alteration in serum bile acids pool (sex and treatment interaction effect) | Lipid metabolism and bile acid metabolism |
| Mock et al. 2017 [9]. | Female rats fed 13% sucrose solutions for 8 weeks | Increased palmitoleic acid in gonadal and retroperitoneal fat pads; higher serum triglyceride | Lipid metabolism |
| Öztürk et al. 2022 [10]. | Wistar male rats fed 10% sucrose in drinking for 3 months | Decreased serum levels of kynurenic acid and kynurenine | Tryptophan metabolism |
| Gariani, Karim, et al. 2016 [11]. | Male C57BL/6J mice were fed with a Western high‐fat and high‐sucrose | Decreased NAD^+^ levels in the liver | Fatty acid oxidation |
| Togo et al., 2019 [12]. | C57BL/6J mice fed liquid (50% by weigh) or solid sucrose for 8 weeks | Elevated hepatic fat | Lipid metabolism |
